# Influence of Heavy Metals and PCBs Pollution on the Enzyme Activity and Microbial Community of Paddy Soils around an E-Waste Recycling Workshop

**DOI:** 10.3390/ijerph110303118

**Published:** 2014-03-14

**Authors:** Xianjin Tang, Muhammad Z. Hashmi, Dongyan Long, Litao Chen, Muhammad I. Khan, Chaofeng Shen

**Affiliations:** 1 College of Environmental and Natural Resource Sciences, Zhejiang University, Hangzhou 310058, China; E-Mails: xianjin@zju.edu.cn (X.T.); hashmi_qau@yahoo.com (M.Z.H.); ldongyan210@gmail.com (D.L.); litao.chen2001@gmail.com (L.C.); 2 Department of Agronomy, University of Agriculture, Faisalabad 38040, Pakistan; E-Mail: khanimran1173@yahoo.com

**Keywords:** e-waste, heavy metals, PCBs, enzyme activity, microbial community, paddy soil

## Abstract

Due to the emerging environmental issues related to e-waste there is concern about the quality of paddy soils near e-waste workshops. The levels of heavy metals and PCBs and their influence on the enzyme activity and microbial community of paddy soils obtained from the immediate vicinity of an e-waste workshop were investigated in the present study. The results indicated that the heavy metal and PCB pollution did not differ significantly with an increase of the sampling point distances (5 to 30 m). The concentration of Cd (2.16 mg**·**kg^−1^) and Cu (69.2 mg**·**kg^−1^) were higher, and the PCB pollution was also serious, ranging from 4.9 to 21.6 μg**·**kg^−1^. The highest enzyme activity was found for urease compared to phosphatase and catalase, and a fluctuating trend in soil enzyme activity was observed in soils from different sampling sites. The microbial analysis revealed that there was no apparent correlation between the microbial community and the pollutants. However, a slight influence for soil microbial communities could be found based on DGGE, the Shannon index and PCA analysis. The present study suggests that the contamination stress of heavy metals and PCBs might have a slight influence on microbial activity in paddy soils. This study provides the baseline data for enzyme activities and microbial communities in paddy soil under the influence of mixed contamination.

## 1. Introduction

Electronic and electric waste (e-waste), defined as end-of-life electronic products, including computers, television sets, mobile phones, transformers, capacitors, wires and cables, are a major environmental concern in China and other countries [1]. According to the report of the United Nations Environment Programme (UNEP), approximately 50%–80% of global e-waste is legally or illegally imported into Asia each year, and about 90% of these e-wastes end up in China where they are illegally recycled in plants or workshops for the profit [2,3,4]. Because many electronic products contain hazardous materials, the primitive extraction processes used, such as strong acid leaching and the open burning of dismantled components, could result in the release of large quantities of toxic metals and organic contaminations into the surrounding soil environment [5,6,7]. The Taizhou region of China is considered the largest e-waste recycling area with both open small e-waste recycling workshops and closed large system plants [5]. Previously, it was found that workshops contribute heavier pollution to the environment in general and particularly those near to the paddy fields compared to the plants [6]. As the quality of the paddy system is important to the agricultural ecosystem and human health, a typical paddy soil from close to an e-waste recycling workshop in the Taizhou region was selected and the quality of paddy soil influenced by the mixed pollution was investigated in this study. 

The paddy field is a unique agro-ecosystem consisting of diverse habitats of microorganisms and other living things. These microorganisms support various important biogeochemical processes occurring in paddy fields. Furthermore, soil microorganisms have predominant roles in the recycling and detoxification of contaminants. Therefore, soil microorganisms can be viewed as a key measure of the contaminated soil quality [8,9,10]. Soil enzymes have also been considered as effective indicators of the general activities of soil microorganisms [11]. Soil enzymes not only serve as an indicator of the soil quality, but also play a significant role in the reduction of PCBs [12]. A similar conclusion was reached concerning the soil near a copper smelter, in which the microbial biomass is negatively affected by the heavy metal stress, and the soil enzyme activity is greatly depressed by the presence of heavy metal contamination [9]. The enzyme activities were also found to be severely inhibited in soils that were contaminated with both PAHs and heavy metals [13]. 

The structure and diversity of microbial communities is a good indicator of the influence of anthropogenic activities on soil ecology. Because only less than 1% of soil bacteria is cultivable by current microbial technologies, the composition of microbial communities by incubation-independent tools was mostly studied to investigate the influence of anthropogenic activities on soil quality [9,14]. Studies have confirmed that the exposure of heavy metals and organic pollutants could influence the microbial communities in soil [9,15,16,17,18,19]. Thavamani *et al.* [13] studied the influence of a mixture of heavy metals and PAHs on soil microbial diversity and enzyme activity and found that soil microorganisms and enzyme activity were reduced with higher levels of mixed contamination. Denaturing gradient gel electrophoresis (DGGE) analysis of 16S rRNA genes has been proved to be a powerful tool to elucidate bacterial community structures [20]. Kim *et al.* [21] used DGGE to study the total soil microbial community profiles in paddy soil that was contaminated by organic pollution. Similarly, Lopes *et al.* [22] investigated the microbial diversity of bulk paddy soil from two rice fields subjected to organic and conventional farming practices used DGGE. 

In our previous study, we reported that simple household workshops contributed more heavy metals and should be given more attention [6]. Serious inorganic and organic pollution and their effects on microbial communities in non-irrigated agricultural soil close to an e-waste recycling workshop have also been investigated [23]. However, the study of paddy soil quality around the e-waste recycling and disposal region is scarce, and the characteristics of the soil microbial communities in the paddy soil around e-waste recycling sites are not well-understood. The main aim of the present study was to analyze whether the mixed contamination would affect the microbial community structure in the paddy soil region and to study the influence through the investigation of soil enzyme activities and soil microbial community profiles.

## 2. Materials and Methods

### 2.1. Soil Sampling

**Figure 1:**
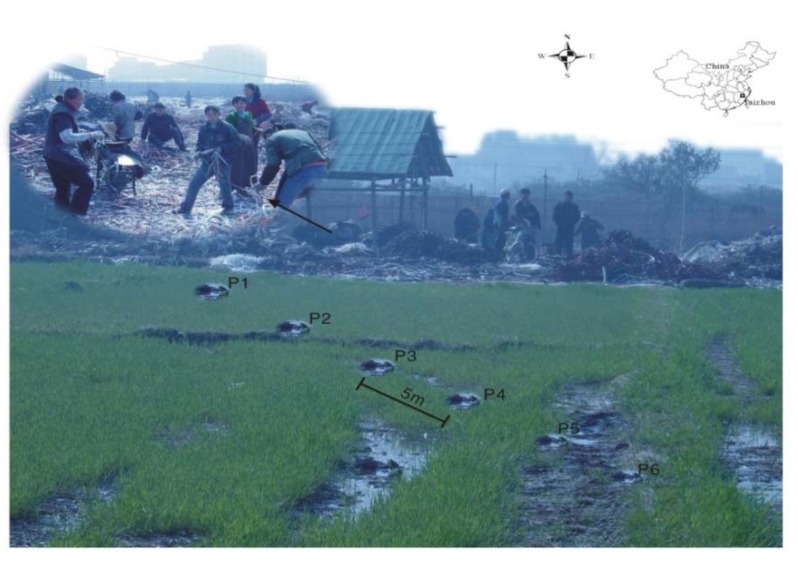
Map of study area and the six sampling points (P1–P6).

A typical workshop, which was located in the Anrong village of Taizhou and was mostly used to dispose of scrap wires and cables, was selected in the present study. The e-waste activities have been going on in this workshop for over 30 years and the techniques used include manual dismantling of fractions, and open burning of wire and cable to recover metals, *etc*. Specially, there was no air or water pollution control measures in this workshop and the generated pollution was released directly into the surrounding environment. A paddy field close to the workshop, as shown in Figure 1, was used to collect the surface soil samples (depth of 0~15 cm). Six soil sampling sites (P1, P2, P3, P4, P5 and P6) were selected at 5 m intervals on a line out from the workshop location, each at a distance of 5, 10, 15, 20, 25 and 30 m from the workshop. At each sampling location, three subsamples (0–15 cm) were collected and pooled together to make one representative sample of about 1,000 g in size. Following the sampling, plants, stones and other items were carefully removed. Soil samples were then stored at 4 and −20 °C for further chemical and biological analysis [23].

### 2.2. Chemical Analysis

Soil pH was determined for 1:2.5 H_2_O suspensions using a glass electrode and soil organic matter content was measured by dichromate oxidation [24], respectively. For the heavy metals analysis, a 0.5 g freeze-dried soil was firstly weighed and digested with a mixture containing hydrofluoric acid and nitric acid/perchloric acid (1:1, v/v) in vessels. Next, the residues was dissolved with hydrochloric acid, filtered and diluted with deionized water. Finally, the concentrations of the metals (Cu, Zn, Pb, Cr, Cd, and Ni) of diluted solution were determined by flame atomic absorption spectrometry (AAS, Solaar-MK II-M6, Thermo Elemental, Fisher Scientific Inc. Toronto, ON, USA) [25,26]. Each soil sample was measured in triplicate and every step of measurements was optimized to meet the quality control standards. For PCBs analysis, 5.0 g freeze-dried soil was extracted in a Soxhlet apparatus for 48 h with a 200 mL mixture of hexane-acetone (1:1, v/v). After concentration, clean-up and evaporation, the concentrations of the PCBs were measured using a gas chromatograph equipped with a ^63^Ni electron capture detector (Agilent 6890N, Agilent Technologies, California, CA, USA) [6,23]. The 13 PCB congeners tested were PCB18, PCB28, PCB31, PCB52, PCB44, PCB101, PCB149, PCB118, PCB153, PCB138, PCB180, PCB170, and PCB194. During the PCBs analysis, two chemicals (2,4,5,6-tetrachloro-*m*-xylene and decachlorobiphenyl) were specially used for determination of PCBs recoveries, which ranged from 85% to 92%.

### 2.3. Soil Enzyme Activity Analysis

Three types of soil enzyme (catalase, urease and phosphatase) were measured in this study. Catalase activity was determined according to Guan *et al.* [27] and Stpniewska *et al.* [28]. Briefly, a 3.0 g dried soil sample was added to a 250 mL flask, then 40 mL distilled water and 5 mL 0.3% H_2_O_2_ were poured into the flask and shaken for 20 min. After shaking, 3 N H_2_SO_4_ was added to terminate the reaction. After filtration, the catalase activity was measured by the titration method with 0.1 N KMnO_4_. The procedure described by Guan *et al.*, [27] and Liang *et al.*, [29] was used to measure the urease activity. Five grams of dried soil and 1 mL toluene were mixed in a 50 mL flask. After 15 min, 20 mL citrate buffer and 10 mL 10% urea solution were added. The reactants were incubated at 37 °C for 24 h. Then, the absorbance of the incubated solution was measured at 578 nm. The method modified from Wang *et al.* [9] was applied to measure the soil alkaline phosphatase activity, a 1.0 g dried sample was incubated with 5 mL borate buffer (pH 10.0), 0.2 mL toluene, and 5 mL disodium phenyl phosphate solution in 50 mL flask at 37 °C for 1 h. Then, the solution was made up to 50 mL volume with 38 °C distilled water and filtered. 2 mL of the filtrate was then transferred into another 50 mL volumetric fiask. Following, 2.5 mL boric acid buffer (pH 9.6), 1.5 mL potassium ferricyanide (2.5%), and 1.5 mL of 4-amino antipyrine (0.5%) were added and carefully mixed. After the solution was diluted to 50 mL and the absorbance of solution was measured at 570 nm after 20–30 min for color development. These three types of soil enzyme were measured three times for each soil sample.

### 2.4. Microbial Counts

To count the viable microbial numbers, a 0.5 g fresh paddy soil sample with three replicates was accurately weighed and serially diluted to obtain a series of dilutions (10^−4^, 10^−5^, 10^−6^, 10^−7^, 10^−8^ and 10^−9^). Then, the dilution liquid was spread on Beef extract peptone medium for bacteria or culture medium of Gause medium No.1 for the isolation of actinomycetes. The plates were incubated for 1–2 days (bacteria) and 4–5 days (actinomycetes) prior to counting the numbers of colony forming units (CFU) of bacteria and actinomycetes [30]. Meanwhile, the water content was determined and the results of microbial counts were expressed as 10^6^/g∙dw∙(dry weight).

### 2.5. PCR-DGGE Analysis

Total soil DNA was extracted and purified from 0.5 g soil using a bead beating method (FastDNATMSPIN Kit for Soil, Bio101 Inc., California, CA, USA) following the manufacturer’s instructions. The isolated the total DNA was conserved in a 1.5 mL EP tube at −20 °C. The primers F357GC: 5’-CGCCCGCCGCGCCCCGCGCCCGGCCCGCCGCCCCCGCCCCCCTACGGGAGGC AGCAG-3’ and R518: 5’-ATTACCGCGGCTGCTGG-3’ were used for the amplification of the bacterial 16S rRNA genes for the DGGE analysis, and the primers F243GC: 5-CGCCCGCCGCGCCCCGCGCCCGGCCCGCCGCCCCCGCCCCGGATGAGCCCGCGGCCTA-3’ and R518 were used for the DGGE analysis of bacteria and actinomycetes, respectively [9,31]. A master cycler gradient was used for the PCR reaction with final volume of 50 μL in 0.2-mL tubes. Taq DNA polymerase (5 units), both primers (25 pmol), 10 × PCR buffer (5 μL), each dNTP (100 mmol) and MgCl_2_ (0.1 mM) were used for the reaction mixture. DNA was amplified by following the steps of the PCR reaction: 5 min initial denaturation at 94 °C, followed by 20 cycles of 94 °C for 1 min, 60 °C for 1 min, 72 °C for 1 min and a final extension period of 72 °C for 7 min. Finally, 1% agarose gel electrophoresis was used to determine the contents of the amplified DNA. For DGGE analysis, the amplified DNA was separated on a 10% acrylamide gel with a linear denaturant gradient range from 35% to 60%. DGGE was performed using 40 μL PCR products in 1×TAE buffer at 60 °C for 5.5 h, and a constant voltage of 150 V (DcodeTM Universal Detection System, Bio-Rad, California, CA, USA). The gels were then stained for 30 min with SYBR-green II and were analyzed for bands [9,32]. 

Digitized DGGE images were analyzed with Quantity One image analysis software (Version 4.0, Bio-Rad) and the similarity of the gel pattern was identified using principal component analysis (PCA). The Shannon index value (H) of the soils was calculated on the basis of the number and intensity of bands present in the DGGE samples. H was calculated as follows: H = ∑(n_i_/*N*) ln(n_i_/*N*), where n_i_ is the intensity for each individual band (i) and *N* is the sum of intensities of all bands in a lane.

### 2.6. Statistical Analysis

All the data were analyzed using SPSS for Windows Release 15.0 (SPSS Inc., Chicago, IL, USA). The probability value of 0.05 was taken as significance. And the data are presented as the mean value for the chemical and biological analyses of each soil sample.

## 3. Results and Discussion

### 3.1. Soil Properties, Heavy Metals and PCBs Analysis

The pH of the six soil samplings (P1, P2, P3, P4, P5 and P6) was 6.3, 5.7, 5.3, 6.1, 5.3 and 5.5, respectively. While the content of soil organic matter was 5.3, 5.1, 5.8, 5.6, 6.7, and 6.5%, respectively. Table 1 shows the concentrations of heavy metals (Zn, Cu, Pb, Cd, Cr, and Ni) in the paddy soil. The results indicate that the average concentrations of Zn, Cu, Pb, Cd, Cr and Ni were 126.5, 69.2, 97.0, 2.16, 69.3 and 34.7 mg**·**kg^−1^, respectively. In the paddy soil region, the concentration of Cd was higher than the Grade III value (1.0 mg**·**kg^−1^) of the National Environmental Quality Standards for soil of China (GB15618-1995); the concentration of Cu exceeded the Grade II value (50 mg**·**kg^−1^), whereas there was a slight pollution of Zn and Pb that exceeded the Grade I value. However, the paddy soil in the Taizhou region was contaminated more by Cd (6.4 mg**·**kg^−1^), Cu (256.4 mg**·**kg^−1^), Cr (33.5 mg**·**kg^−1^), Pb (67 mg**·**kg^−1^) and Zn (209.9 mg**·**kg^−1^) [33]. Interestingly, the lowest concentrations of heavy metals (especially, Zn and Cu) were measured in P1 sample which is closest to the workshop and the heavy metals pollution did not decrease with the distance from the pollution source. A clear decrease of heavy metals with the distance from the pollution source in dry land soil could be shown in our previous research [23]. We suppose the main reason might be due to the special nature of the paddy soil samples. The paddy ecosystem always remains under the influence of irrigation and ploughing which might be responsible for the different levels of heavy metals contamination in paddy soil compared to dry land soil. Therefore, no clear decrease of heavy metals with the distance from the pollution source in paddy soil was found in the present study. However, the results presented in this study suggest that the dumping of a large amount of unsalvaged e-waste may discharge metals in the paddy soil directly. The flooding of the paddy soil with water which receives the discharging of the waste solution also increases the metal concentration. Further, the receiving of wet or dry depositions accounts for an important reason for metal pollution of the paddy soil [33]. In addition, the low contents of Pb and Cr might be a reflection of the effects of different types of e-waste processing activities and their resultant discharge.

**Table 1:** Heavy metals content in paddy soil collected from Taizhou region (mg**·**kg^−1^**·**dw).

Sampling No.	Distance (m) ^a^	Zn	Cu	Pb	Cd	Cr	Ni
P1	5	95.44 ± 9.78 a	37.17 ± 3.07 a	87.22 ±7 .69 a	1.83 ± 0.28 ab	60.17 ± 5.33 a	30.77 ± 2.39 a
P2	10	126.53 ± 10.17 b	81.14 ± 0.64 b	92.16 ± 7.82 ab	2.08 ± 0.33 abc	68.17 ± 4.12 b	32.70 ± 1.17 ab
P3	15	134.50 ± 8.71 bc	78.26 ± 0.67 c	107.10 ± 9.40 c	2.56 ± 0.59 c	74.18 ± 1.11 b	35.58 ± 0.76 c
P4	20	129.00 ± 9.26 bc	71.36 ± 0.55 d	99.54 ± 6.15 abc	2.57 ± 0.34 a	69.04 ± 3.60 b	35.37 ± 1.84 bc
P5	25	131.34 ± 4.12 bc	68.59 ± 0.57 d	92.22 ± 4.30 ab	1.71 ± 0.24 b	71.11 ± 3.18 b	34.89 ± 0.66 bc
P6	30	142.00 ± 12.73 c	78.94 ± 0.14 bc	103.98 ± 3.61 bc	2.23 ± 0.19 abc	73.53 ± 1.84 b	38.93 ± 1.60 d

Notes: ^a^ Distance from the e-waste recycling workshop. Values in each column followed with different letters indicated significant (*p* ≤ 0.05) differences among different soil samples, and values represent means ± standard deviation.

**Figure 2:**
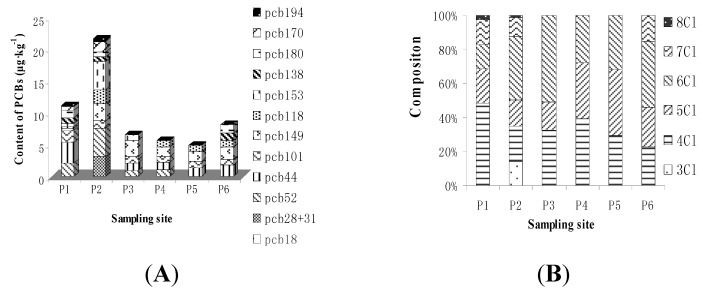
Characterization ((**A**) dry weight basis) and composition (**B**) of PCBs in paddy soil samples.

The concentrations of 13 PCB congeners were measured by gas chromatography. As shown in Figure 2, the total contents of PCBs ranged from 4.9 to 21.6 μg**·**kg^−1^ with an average of 9.70 μg**·**kg^−1^, therefore, the pollutions of the sample sites (P1–P6) as at a relatively low level compared to those found in previous studies such as Shen *et al*. [6] with a mean concentration of 41.0 μg**·**kg^−1^, and Tang *et al.* [7] with a mean concentration of 70.6 μg**·**kg^−1^, although the number of PCB congeners were different. The results suggest that the low level of PCBs in all sites may be due to the lower deposition of PCBs in paddy area from the e-waste recycling workshop [6,34]. The compositions of PCBs homologues in each sample are also presented in Figure 2. Since PCBs from the same sources exhibit similar PCBs compositions, the similar compositions of the PCBs revealed that the source of the PCBs must be similar. Shen *et al.* [6] also found similar profiles of those PCB congeners in soil from different plants and workshops in China, and suggested they could come from similar sources. In the present study among PCB congeners, lesser chlorinated congeners, including tri-CBs, tetra-CBs and penta-PCBs, were the most prevalent homologues, accounting for 71.6%–78.8% of the total concentrations of PCBs. This may be due to the low chlorinated congener’s behavior as they transported more easily than the high chlorinated congeners [35]. The profiles of transformer oils, which are produced and used in China, have a large proportion of lower molecular weight congeners, particularly tri-PCB, followed by tetra-PCB [36]. This statement was demonstrated in transformer oil samples, which showed that insulating oils of locally produced transformers mainly contained lower chlorinated PCBs [37,38].

The correlation matrix among heavy metals and PCBs in paddy soil was investigated. Statistically significant correlations could be found between Cd and Pb (r = 0.815, *p* < 0.05) and Cu with Zn (r = 0.922, *p* < 0.01), which indicated that these compounds might have a similar source. However, no significant correlation was found among heavy metals and PCBs in paddy soil in the present study. The correlation results were different from our previous study in which a strong correlation among Cu, Pb and PCBs could be found in paddy soil [7], suggesting the complex sources of pollutants or the various e-waste recycling materials in the study area. 

### 3.2. Enzyme Activities

All of the enzyme activities showed fluctuating results in the present study (Table 2). The enzyme activities followed the order: urease > phosphatase > catalase. The higher activities of urease followed by phosphatase, compared to the catalase, may be due to their sensitivities to heavy metals and PCBs [8,39]. Hu *et al.* [40] found that the urease activity was higher than the phosphatase or catalase activity in soil influenced by mixed contamination of heavy metals. The authors suggested that the urease activities were more sensitive to the mixed contamination than the other two enzymes based on the EC50 values measurement. The three types of enzyme activity at P1 (4.27 mL**·**g^−1^, 182.56 mg**·**kg^−1^**·**24∙h^−1^ and 66.65 mg**·**kg^−1^**·**h^−1^, respectively) was lower than the other sites (P2–P6). Generally, a fluctuating trend in catalase, urease and phosphatase activity was obtained in soil from different sampling sites. However, a slowly increasing trend in catalase and urease activity could be seen from P1 to P6 except P3. The phosphatase activity also increased slowly from P1 to P5. There are considerable amount of evidences documenting a decrease in the soil enzyme activity as a result of exposure to heavy metal or mixed contamination [41,42]. In addition, the bioavailability of metals and organic pollutants to microbes at different points could also influence the soil enzyme activity [13]. Furthermore, Andreoni *et al.* [43] have stated that relatively low soil enzyme activity could be explained by the interference of high Cu contents in Italian soil samples. Since the paddy soil around the workshop had a low level of heavy metals and PCBs based on our investigation, the fluctuating enzyme activity might suggest that the low concentration of mixed pollution emissions might have no pronounced effect on enzyme activity in paddy soil.

**Table 2:** Soil enzymes activities in paddy soil in different distances.

Sampling No.	Distance (m)	Catalase (mL·g^−1^·dw)	Urease (mg∙kg^−1^·24∙h^−1^∙dw)	Phosphatase (mg∙kg^−1^·h^−1^·dw)
P1	5	4.27 ± 0.17 a	182.56 ± 12.82 a	66.65 ± 11.05 a
P2	10	5.97 ± 0.18 b	290.93 ± 20.17 b	100.27 ± 7.46 b
P3	15	6.28 ± 0.26 bc	385.35 ± 26.24 c	186.98 ± 33.56 cd
P4	20	5.79 ± 0.13 b	290.74 ± 1.61 b	222.90 ± 19.87 ce
P5	25	6.72 ± 0.07 c	309.18 ± 7.26 b	231.49 ± 16.88 e
P6	30	6.75 ± 0.75 c	339.96 ± 14.79 d	189.32 ± 17.20 d

Notes: Values in each column followed with different letters indicated significant (*p* ≤ 0.05) differences among different soil samples and values represent means ± standard deviation.

### 3.3. Microbial Counts

The number of bacteria and actinomycetes in paddy soil collected from different sampling sites were determined by plate counting and are given in Table 3. With an increase of distance, the bacteria counts increased slowly from the nearest site (P1) to the farthest site (P6), except for P5. A good relationship was observed between the bacteria and the distance from the disposing and recycling site. These results are consistent with the soil enzyme activities. On the contrary, the actinomycetes counts fluctuated from site to site and showed anomalous information. It was found that there was no significant correlation between the counts of bacteria and actinomycetes and contamination in the paddy soil. However, a different conclusion was reached in a study conducted by Wang *et al.* [9], in which the microbial biomass carbon was negatively affected by the elevated metal levels and was closely correlated with heavy metal stress in soil from a copper-zinc smelter.

**Table 3:** The number of microbes (bacteria and actinomycetes) in different sampling sites.

Sampling No.	Distance (m)	Bacteria (10^6^/g·dw)	Actinomycetes (10^6^/g·dw)
P1	5	0.40 ± 0.06 ab	0.19 ± 0.01 ab
P2	10	0.38 ± 0.07 ab	0.32 ± 0.03 a
P3	15	0.54 ± 0.19 ac	0.23 ± 0.02 ab
P4	20	0.59 ± 0.16 ac	0.12 ± 0.01 b
P5	25	0.19 ± 0.16 b	0.22 ± 0.12 ab
P6	30	0.69 ± 0.09 c	0.21 ± 0.01 ab

Notes: Values in each column followed with different letters indicated significant (*p* ≤ 0.05) differences among different soil samples, and values represent means ± standard deviation.

### 3.4. Microbial Community Structure Analysis

**Figure 3:**
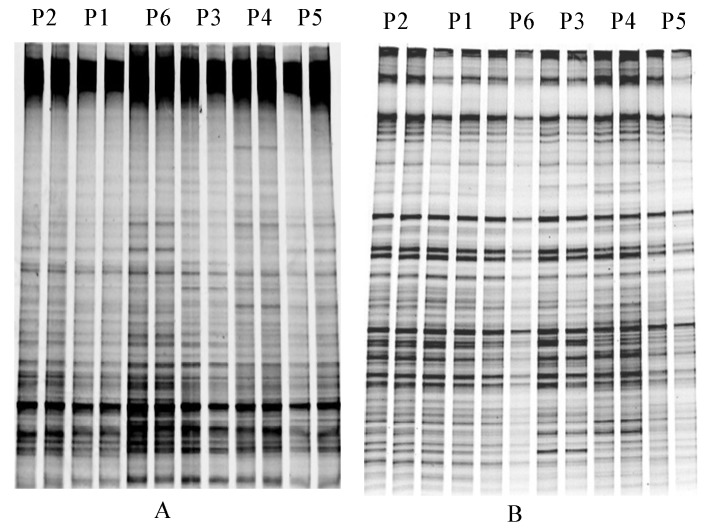
DGGE profiles of soil bacteria (**A**) and actinomycetes (**B**) communities from different soil samples.

**Figure 4:**
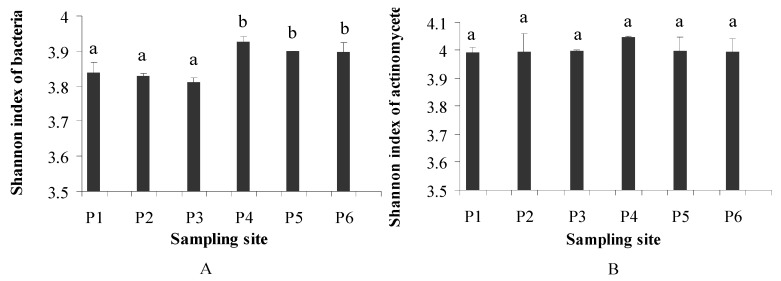
Shannon diversity indexes of bacteria (**A**) and actinomycetes (**B**) in different soil samples. (Different letters in the column indicated significant difference, *p*≤ 0.05).

The PCR-DGGE method was performed to analyze the microbial community structure influenced by the heavy metals and PCBs in paddy soil. Figure 3 shows the DGGE band patterns of the bacteria and actinomycetes communities in the paddy soil. Generally, no difference was found between the bands at the different sampling sites, which indicates that the microbial (*i.e*., bacteria and actinomycetes) community structure was very similar in all samples taken from the contaminated paddy soil. The results suggested that the microbial community structures of paddy soil from different points were not seriously influenced by heavy metals and PCBs. Several factors might contribute to the similar structure of microbial communities of paddy soil, such as a low level of contamination [39] and the co-abundance of different organisms in the paddy soil [44]. The same soil water content may also hinder the clear effects of mixed contamination on the bacterial community structure. The DGGE gels were further analyzed by the Shannon index, and the results are shown in Figure 4. The bacterial Shannon index of P1 was similar to P2 and P3, whereas P4 exhibited the highest index value and was close to P5 and P6. No significant differences were found among the Shannon index values of the actinomycetes in the paddy soil among the six sampling sites. Overall, the change in the microbial diversity of the six soil samples from the paddy soil region was indistinctive (Figure 4). The first three points (P1–P3) showed loss in Shannon index values and this might be due to the relatively high levels of PCBs (P1 and P2) and heavy metals (*i.e*., Cu, Pb and Cd in P3). The similar loss in Shannon index value had been found in a study by Thavamani *et al.* [13] in those sites which had high pollution of heavy metals and PAHs. 

**Figure 5:**
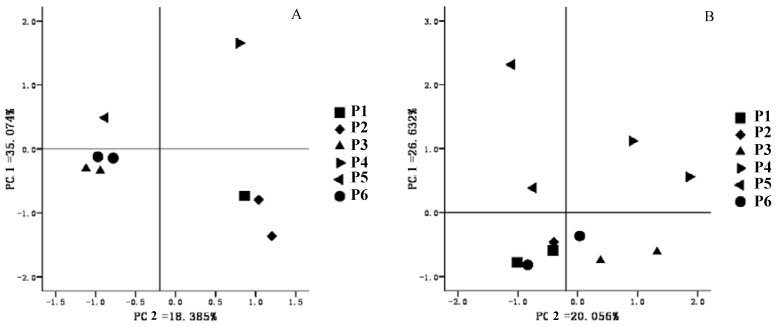
PCA analysis results based on DGGE profiles of paddy soil samples ((**A**): bacteria, (**B**): actinomycetes).

PCA of soil fingerprints (DGGE bands) is a useful tool to detect shifts in the microbial composition of mixed contaminated soil [9]. PCA results based on the DGGE profiles of paddy soil are shown in Figure 5 and reveal that the microbial community structure of P1 was similar to that of P2 but different from that of P3, P4, P5, and P6. For the P1 and P2, there was no significant difference of four heavy metals except Zn and Cu. PCBs levels in these two sites (12.1 and 21.6 μg**·**kg^−1^, respectively) were also obviously higher compared to other sites. It seems that the similar levels of mixed contamination especially PCBs in these two sites might possibly contribute to the similar microbial community structure in soil, although there were other various factors such as the co-abundance of different organisms and the same soil water content in paddy soil as we discussed above. The differences in bacterial community structure were not significant among P3, P5 and P6. While PCA results for the actinomycetes revealed that there was minor difference in the community structure between two consecutive sites (P3–P6, Figure 5B). Over all, there was no good relationship to be found between the microbial parameters including enzyme actives and DGGE results with mixed contaminations of heavy metals and PCBs in our study. Although Wang *et al.* [9] suggested that there were negative correlations among microbial biomass, enzyme activity, and metals in soil with different distances from a smelter. The PCR-DGGE analysis results also indicated that soil-available metals concentrations were well correlated with the relative abundance of *Comamonadaceae*, *Moraxellaceae*, and other bacterial communities in soil [45]. Since soil microbial community structure is an important component in the regulation of the soil microbial activity [42], and the toxicity effects of chemical pollutants on soil microbial activity have been confirmed [46], the microbial community shifts in the present study suggests that the contamination stress of heavy metals and PCBs might pose a slight influence on microbial activity in paddy soil. Due to the emerging environmental issues of e-waste, the potential threats of mixed contamination to the quality and health of paddy soil should be further studied.

## 4. Conclusions

This study was conducted to investigate the influence of heavy metals and PCBs on the microbial community structure in paddy soil from Taizhou, China. The results indicated that the pollutants had no significant influence on the microbial population among the different sampling points. Different enzymes activities in the paddy soil were detected, and a fluctuating trend in catalase, urease and phosphatase activity was obtained in soils from different sampling sites. However, the results based on DGGE, the Shannon index and PCA analysis revealed that the soil microbial community structure was slightly influenced in the paddy soil with heavy metals and PCBs contamination. The present study suggests that the contamination stress of heavy metals and PCBs might have a slight influence on microbial activity in paddy soil. Due to the emerging environmental issues of e-waste, the potential threats of mixed contamination to the quality and health of paddy soil should be further studied.
